# Outbreak of Oropouche virus in frontier regions in western Amazon

**DOI:** 10.1128/spectrum.01629-23

**Published:** 2024-02-07

**Authors:** Hillquias Monteiro Moreira, Gabriella Sgorlon, Jackson A. S. Queiroz, Tarcio P. Roca, Jessiane Ribeiro, Karolaine S. Teixeira, Ana Maísa Passos-Silva, Adrhyan Araújo, Nadson Willian Felipe Gasparelo, Alcione de Oliveira Dos Santos, Celina Aparecida Bertoni Lugtenburg, Rosemary Aparecida Roque, Juan Miguel Villalobos Salcedo, Dhelio B. Pereira, Deusilene Vieira

**Affiliations:** 1 Laboratório de Virologia Molecular, Fundação Oswaldo Cruz Rondonia - FIOCRUZ/RO, Porto Velho, Rondonia, Brazil; 2 Programa de Pós-Graduação em Biologia Experimental, Universidade Federal de Rondonia - UNIR, Porto Velho, Rondonia, Brazil; 3 Centro de Pesquisa em Medicina Tropical, CEPEM, Porto Velho, Rondonia, Brazil; 4 Laboratório Central de Saúde Pública de Rondonia, LACEN/RO, Porto Velho, Rondonia, Brazil; 5 Instituto Nacional de Pesquisas na Amazônia, INPA, Manaus, Amazonas, Brazil; Emory University School of Medicine, Atlanta, Georgia, USA

**Keywords:** Oropouche, OROV, epidemiology, genomic surveillance, phylogeny

## Abstract

Oropouche virus (OROV) is characterized as a re-emerging arbovirus of great concern for public health, being responsible for several outbreaks of acute fever identified in Latin American countries, registering more than half a million reported cases. The incidence of reports of this virus is intrinsically favored by environmental conditions, in which such characteristics are related to the increase and distribution of the vector population to areas of human traffic. Moreover, there is a problem regarding the lack of diagnosis in Brazil that aggregates the success of the etiologic agent. Thus, by means of molecular techniques, we identified 27 positive cases of the OROV circulating in border locations in western Amazon, with 44.44% (12/27) of the cohort characterized as infected individuals with reported symptoms, mainly ranging from fever, myalgia, and back pain. Among the positive samples, it was possible to obtain a total of 48.14% (13/27) samples to analyze the S and M segments of Oropouche, which showed similarities among the Brazilian sequences. Thus, it was possible to verify the circulation of the OROV in Rondonia and border areas, in which the tracking of neglected arboviruses is necessary for the genomic surveillance of emerging and re-emerging viruses.

**IMPORTANCE:**

The western Amazon region is known for outbreaks of acute febrile illnesses, to which the lack of specific diagnostics for different pathogens hinders the management of patients in healthcare units. The Oropouche virus has already been recorded in the region in the 1990s. However, this is the first study, after this record, to perform the detection of individuals with acute febrile illness using a screening test to exclude Zika, dengue, and chikungunya, confirmed by sequencing the circulation of the virus in the state of Rondonia and border areas. We emphasize the importance of including diagnostics for viruses such as Oropouche, which suffers underreporting for years and is related to seasonal periods in Western Amazon locations, a factor that has a direct influence on public health in the region. In addition, we emphasize the importance of genomic surveillance in the elucidation of outbreaks that affect the resident population of these locations.

## INTRODUCTION

Oropouche virus (OROV), belonging to the *Peribunyaviridae* family of the *Orthobunyavirus* genus (1) is considered a re-emerging arbovirus responsible for acute febrile outbreaks in tropical regions of Central and South America (2, 3). From 1950 to the present day, more than half a million infections have been registered in the Brazilian Amazon region and Latin America, including countries such as Peru, Tobago and Trinidad, and Panama (2).

OROV is the etiological agent of Oropouche fever, characterized as a zoonosis, transmitted to humans through blood meal mainly by the vector *Culicoides paraensis* (4, 5) and *Culex quinquefasciatus* as a secondary urban vector (6); however, transmission by other specimens of the genera *Culex* and *Aedes* is discussed, which are found in high densities in wild and urban areas (6, 7).

The clinical manifestations caused by OROV consist of acute fever, accompanied by headache, myalgia, arthralgia, anorexia, dizziness, chills, nausea, vomiting, diarrhea, epigastric pain, photophobia, and retro-orbital pain (8). However, because they are non-specific and very similar to the symptoms caused by the more widespread arboviruses such as dengue (DENV), Zika (ZIKV), and chikungunya (CHIKV) (9), screening and diagnosis of Oropouche fever become a challenge (10).

Between the years 2018 and 2021, the state of Rondonia becomes the second in the deforestation ranking of the Brazilian Legal Amazon. In addition, there is a large concentration of deforestation area in undesignated public forests in the border region between Amazonas, Acre, and Rondonia, known as AMACRO, where outbreaks of Oropouche fever have been reported (11–13).

Environmental factors such as these are described as influential in altering the habitat of OROV reservoirs and vectors (14). Furthermore, the climatic factors inherent to the region collaborate to viral dissemination, potentiating the emergence and re-emergence of several arboviruses in the region, causing a major public health problem (8).

Considering that the laboratory diagnosis for arboviruses is routinely directed to DENV, ZIKV, and CHIKV, the real understanding of the epidemiological context of other acute febrile diseases circulating in the region is limited. In 2020, a study conducted for the molecular screening of DENV showed 95.51% (288/308) of negative samples for malaria, dengue, Zika, and chikungunya (15), indicating the need for the diagnosis and screening of other widespread arboviruses. It is understood that surveillance of arboviruses is extremely important, especially in monitoring the distribution of febrile cases in the country and in preventing new outbreaks. However, despite the recurrence of infections by OROV, epidemiological surveillance of the disease is a major challenge. Therefore, the objective of this study was to describe an outbreak occurring in the border regions between the states of Amazonas and Rondonia through molecular screening for OROV in individuals with acute febrile conditions in western Amazon.

## RESULTS

**Fig 1:**
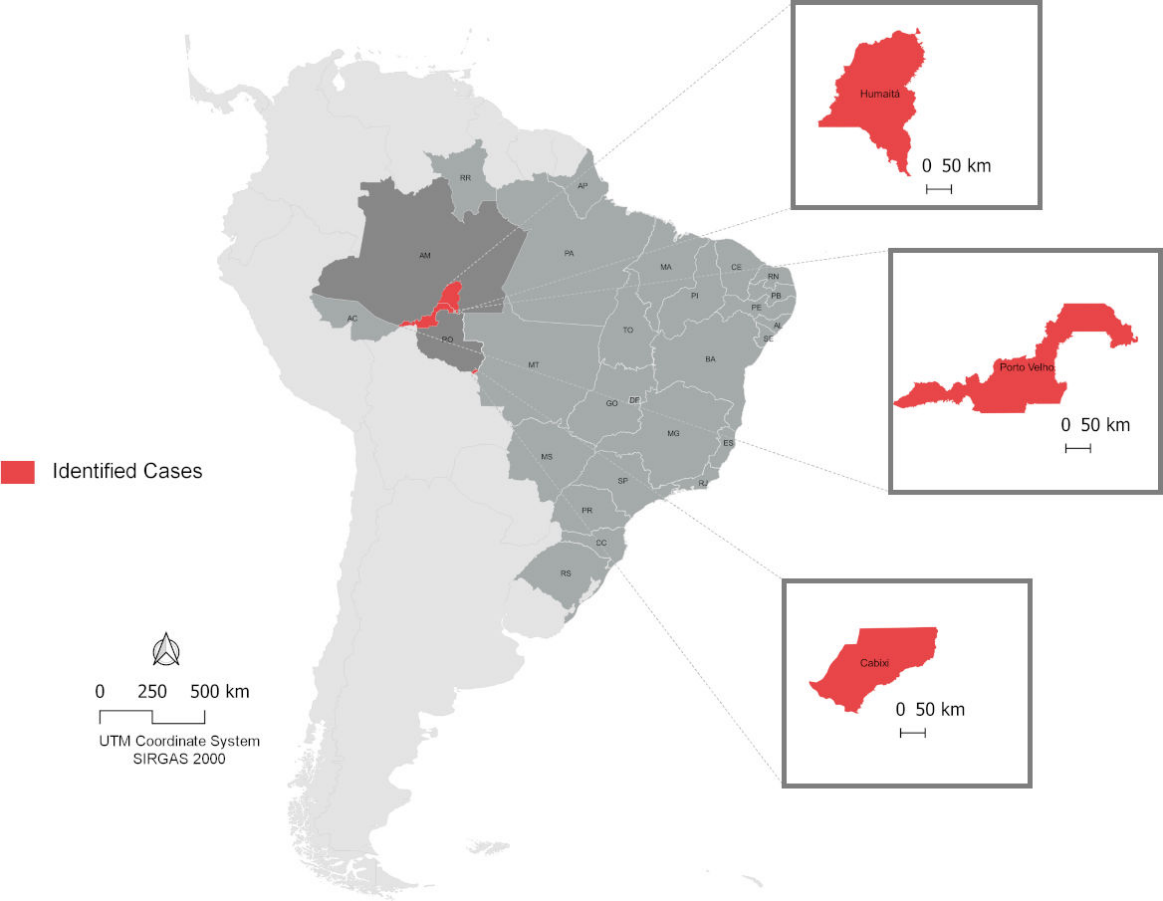
Identification of municipalities in the states of Rondonia and Amazonas that had confirmed cases of Oropouche. Subtitle: Geographic distribution of the municipalities in Rondonia and Amazonas showing the location on the map where the cases were identified. The geographic coordinates of the locations described are Porto Velho, Brazil: 08° 45′ 43″ S, 63° 54′ 14″ W; Cabixi, Brazil: 13° 29′ 52″ S, 60° 33′ 15″ W; and Humaita in Amazonas, Brazil: 7° 30′ 22″ S, 63° 1′ 38″ W.

Among the samples from patients with acute fever tested in this study, 351 were negative for ZIKV, DENV (Serotypes 1 to 4), CHIKV, malaria, and Mayaro. Of these, 7.69% (27/351) were positive for OROV in the cities of Porto Velho, Cabixi, and Humaita (Fig. 1).

Although 62.96% (17/27) of the cases belonged to the city of Humaita in Amazonas State, there was no variation reported as to the point location of the individuals. In contrast, the three places of the cases in Rondonia were reported, observing the accuracy as to the location of the infections described and their geographical positions in the state, where seven cases were detected in the southern and eastern regions in Porto Velho and two cases in Cabixi.

Regarding the dates of identification of OROV, it was found that among the locations in Rondonia, Cabixi recorded the oldest dates of infection among the samples tested, both described in mid-March 2022. In Porto Velho, the state capital, positive cases were cataloged between January and February 2023, presenting in common, the period of high rainfall in the region. In the municipality of Humaita, cases were identified from November 2022, with current records in January and February 2023.

With respect to the epidemiological data of this cohort, 59.26% (16/27) of the infected individuals were male and 40.74% (11/27) were female, with a median age of 43 years (min: 15 and max: 73). There were clinical records of only 44.44% (12/27) of the cases in the study (Table 1), among which, only two cases reported travel to rural areas 15 days before the collection date. The most frequent symptoms in the individuals were fever, headache, back pain, and, to a lesser extent, intense arthralgia and arthritis.

**TABLE 1:** Epidemiological and clinical characteristics of positive cases for Oropouche

Features	Value
Male/female	16/11
Age (years, mean ± SD)	43 ± 17.68
**Symptoms**	***n* (%**)
Fever	12 (100)
Headache	11 (92)
Myalgia	7 (58)
Back pain	6 (50)
Nausea	4 (33)
Retro-orbital pain	3 (11)
Arthralgia	2 (17)
Arthritis	1 (8)
Petechiae	0 (0)
Vomit	0 (0)
Conjunctivitis	0 (0)
Exanthema	0 (0)

**Fig 2:**
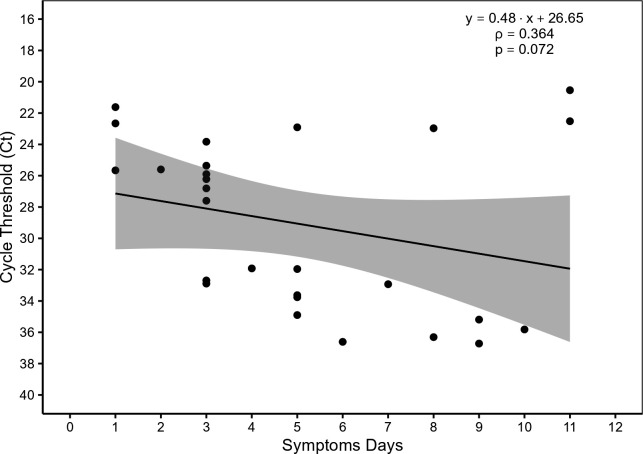
Correlation between detection of OROV-positive samples by RT-qPCR and symptoms days. Subtitle: Cycle threshold (Ct) values detected in 27 positive samples by RT-qPCR for Oropouche viral RNA and symptoms days.

The positive samples had wide dispersion for cycle threshold (Ct) values, which showed an interquartile median of 31.92 (SD = 5.56) and that 51.85% (14/27) were detectable with Ct ≤28. The days of symptoms showed a mean of 5 days (SD = 3.04) (Fig. 2).

**Fig 3:**
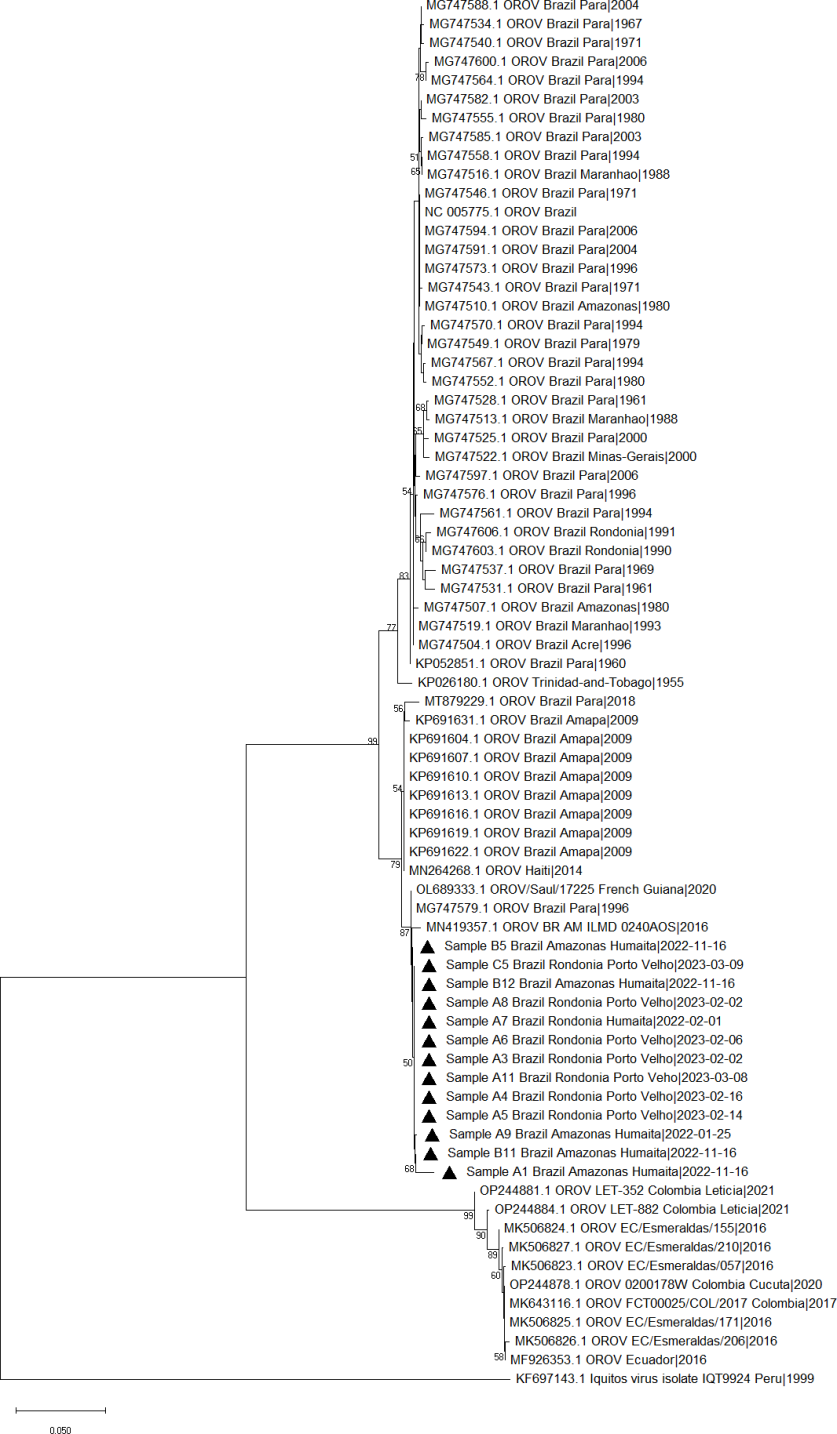
Maximum likelihood phylogenetic tree of segment M showing sequences retrieved from GenBank (*n* = 66) and study samples represented as black triangles. The bootstrap values are contained in the branches.

**Fig 4:**
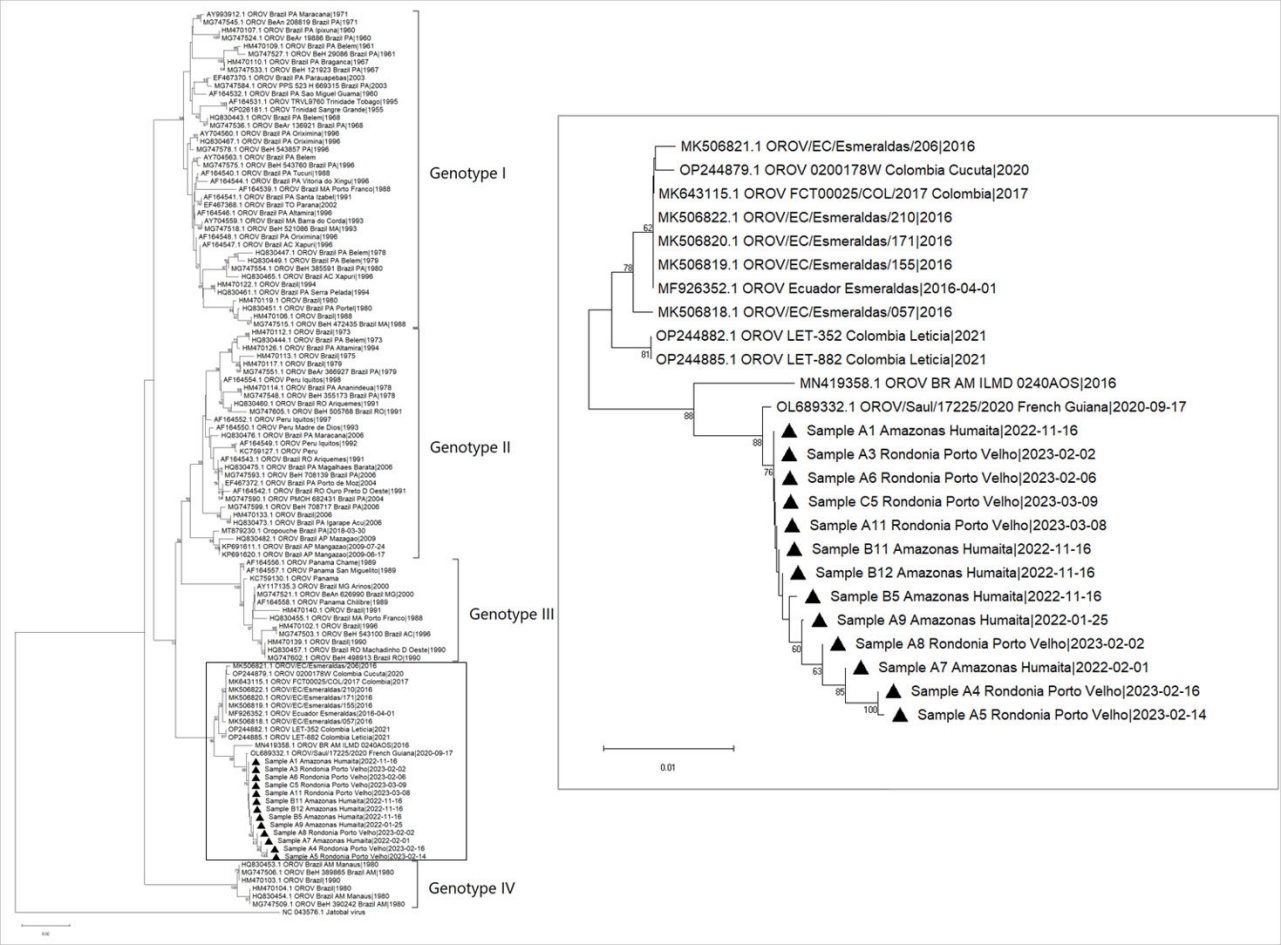
Maximum likelihood phylogenetic tree of segment S showing sequences retrieved from GenBank (*n* = 105) and study samples represented as black triangles. The bootstrap values are contained in the branches.

Samples with Ct ≤28 were selected for sequencing. The partial region of the S and M segments was successfully sequenced in 48.14% (13/27) samples (Table S1). Phylogenetic analysis of the segment M showed the clustering of all the study samples into a same clade and shared a similarity with two sequences from Brazil (Fig. 3). The phylogenetic analysis of the segment S showed a cluster containing samples from Colombia (16, 17) and Ecuador (18); however, the isolates in this study were more closely related to a sequence from French Guiana (unpublished) and Manaus (19), a Brazilian city (Fig. 4).

## DISCUSSION

Brazil has annual peaks of endemicity of different arboviruses, such as Zika, chikungunya, and dengue, in different regions of the country, and other viruses are also reported to cause sporadic outbreaks in the Brazilian Amazon, for example, the Mayaro and Oropouche viruses (20–22).

In this study, we analyzed 351 samples of acute febrile cases tested for ZIKV, DENV, and CHIKV, identifying the circulation of OROV among these individuals in seasonal periods in western Amazon. In Rondonia, acute febrile patients with a negative diagnosis for ZIKV, DENV, CHIKV, and malaria have become an observable and worrisome aspect of public health, due to the large circulation of several arboviruses combined with the scarcity of broad-spectrum diagnostics for distinct species (23).

OROV has been responsible for several occasional febrile outbreaks in the Brazilian Amazon, and it is estimated that between 1961 and 1996, about 357,000 individuals were infected with the Oropouche virus (4), including the states of Amazonas (1980) (24), Maranhão and Goias (1988), and Rondonia (1991), which alone presented approximately 91,000 cases (11). Another four outbreaks were also registered in Belem, the capital of the state of Para located in the northern region, between the years 1961 and 1980, with approximately 131,000 cases (11). After 26 years, in 2006, a resurgence of the virus was reported with about 17,000 cases in Para state (11, 25). The OROV is a major public health concern due to morbidity and reports of emergence and re-emergence in Central and South America (26–28).

Several factors contribute to the circulation of OROV in the region, such as the Amazonian winter, which is characterized by increased pluviometer index, with hot and moist climate from December to May. This climate variation may become a factor of strong influence on the increase in the population of Oropouche virus vectors, as reported in a recent study in Florida, USA, a state with a subtropical climate, which discriminated the seasonal behavior of flies of the genus *Culicoides* and reported increased captures of specimens in spring (29). However, in Brazil, no recent studies described the behavior of this vector in the Amazon region concerning climatic variation. In this study, the months of OROV detection include November, January, February, and March, which are considered to have high rainfall (160–320 mm) in the Amazon (30).

Another aspect found for the geographic expansion of the OROV infection is deforestation, which acts as an important factor for transmission by the vector (31, 32). The localities of the positive cases for OROV in Rondonia in this study are located in the peri-urban areas of the municipalities, with a distribution close to forest areas where contact with these areas has proven to be a predictive factor for viral infection of OROV (33). Increasing population density through urbanization and frequency of exposure of humans to mosquitoes consequently increase the patterns of virus-vector-host interactions (32, 34–36). Simultaneously, in the last few years, agricultural activity, cattle ranching, and widespread deforestation have intensified in response to the demands of development. It is important to note that the state of Rondonia is in the fourth place in the Amazon deforestation ranking, with 1,380.72 km^2^ of the area devastated in 2022 alone; whereas, the state of Amazonas is in the second place, with 2,976.67 km^2^ of the area lost (13). The two states add up to 4,357.39 km^2^ of a lost area in 2022 alone.

Concerning the age range, authors report that females were in a higher percentage of OROV-positive cases than children under 15 years of age (20, 34). In this study, it was possible to observe a different profile, in which males represented the largest number of infected individuals, and the age had a median of 42.7 years with no reports of infection in individuals under 15 years of age.

The clinical manifestations are similar to the main known arboviruses, such as dengue, Zika, chikungunya, and yellow fever, characterized by the presence of an acute febrile condition, with clinical symptoms such as fever, headache, myalgia, arthralgia, and skin eruptions, and may evolve to meningitis or encephalitis (4, 9), which complicates the establishment of a clinical prediction model for the diagnosis of OROV based on signs and symptoms alone (37, 38). Although a peculiarity of OROV has been observed through reports of recurrence of symptoms, it is not noticed in other arboviruses (39). Among the cases isolated in this study, no severe cases of the disease were observed; however, a clinical profile that corroborates the other findings in Brazil in the last 20 years was observed (25, 40). It is important to note that some OROV genotypes are related to these neurological manifestations and have been reported circulating in municipalities of the state of Rondonia (41, 42).

Until 2015, routine diagnosis for arboviruses was restricted to DENV, and with the emergence of CHIKV and ZIKV in the country, especially with ZIKV, which is associated with cases of microcephaly, there was an urgent need for the inclusion of these viruses in routine diagnosis to reduce the impact caused in public health (43, 44). The re-emergence of OROV in the Amazon region underscores the importance of developing and implementing molecular assays for the diagnostics of neglected viral diseases. In Brazil, currently, there are no molecular assays registered by the Health Surveillance Agency (ANVISA) that can be used for differential diagnosis of OROV, despite its potential to cause meningitis already reported in the country (45) and be considered, after DENV, the second most common arbovirus in the Brazilian Legal Amazon (46).

Thus, given the facts exposed, it is believed that other regions may also be subject to outbreaks of the disease because Brazil presents favorable climatic and environmental conditions for the dissemination of this arbovirus (47), which underscores the importance of genomic surveillance in screening for emerging and re-emerging arboviruses.

Analysis of the segments S and M showed a closer phylogenetic relationship with a sequence from Manaus, AM (MN419357.1 and MN419358.1) in both segments (19). However, each of these segments showed different possible routes of introduction.

The formation of an independent clade for segment S, together with sequences from an outbreak in Esmeraldas, Ecuador (2016) and Leticia, Colombia (2021), suggests a transmission route of international origin (16, 48). Notably, for segment M, a more distant relationship is observed with these same isolates, with the formation of an independent clade with Brazilian sequences. These sequences probably correspond to the same viral strain that caused outbreaks in specific cities and continued to circulate in neighboring regions in the western Amazon, subsequently causing the virus to re-emerge in the states of Rondonia and Amazonas recently.

Although reassortments are known to occur in the evolution of Orthobunyavirus (49, 50), we discarded the possibility of reassortment, because all the isolates in this study show a high degree of similarity to an isolate from Manaus during an outbreak in 2016, both for the M segment and for the S segment.

In conclusion, the detection of OROV in the study regions highlights the importance of genomic surveillance of acute febrile illness cases and the need for expansion of specific diagnosis for the second major arboviruses of public health concern, besides alerting to a possible spread of the virus in other regions of the states of Rondonia and Amazonas located in western Amazon.

## MATERIALS AND METHODS

### Study site and biological samples

Samples were collected from individuals included in the study, with acute febrile illness preferentially with 5 to 7 days of symptoms collected in Unidades Básicas de Saúde (UBS) or Unidades de Pronto Atendimento (UPA) in 28 municipalities in the state of Rondonia and in one border municipality belonging to the state of Amazonas from January 2022 to March 2023. The study excluded indigenous people, pregnant women, and patients who did not give written consent. The samples were initially screened for ZIKV, DENV, and CHIKV using the Biomol ZDC Kit (Instituto de Biologia Molecular do Paraná, Brazil), following the manufacturer’s instructions at the Laboratório Central do Estado (LACEN/RO). All biological samples tested were sent to the Laboratório de Virologia Molecular da Fundação Oswaldo Cruz Rondonia (FIOCRUZ/RO) for molecular analysis of Oropouche and Mayaro. Written informed consent was obtained from each participant and/or their legal guardian(s) before sample collection, and all experiments were performed by relevant guidelines and regulations.

### Viral RNA extraction

Viral RNA from the samples was isolated from 140 µL of serum using the QIAamp Viral RNA Mini Kit (QIAGEN, Hilden, Germany) according to the manufacturer’s instructions and was eluted in 60 µL of elution buffer.

### Molecular detection for OROV

Samples were tested in a duplex RT-qPCR adapted from NAVECA et al. (51) for the detection of Mayaro and Oropouche viruses. The reactions were performed in a QuantStudio 7 Pro Real-Time PCR System (Thermo Fisher Scientific, Massachusetts, USA), with the following reaction profile: 50°C for 5 minutes, 95°C for 20 seconds, 45 cycles of 95°C for 3 seconds, and 60°C for 30 seconds. The result was considered positive when the cycle threshold was ≤38 for the analyzed virus.

### Reverse transcription and conventional PCR

RNA extracted from positive samples was subjected to reverse transcription for complementary DNA (cDNA) synthesis using SuperScript IV RT enzyme (Thermo Fisher Scientific, Massachusetts, USA) associated with 0.5 µg of random primer, according to the manufacturer’s instructions.

The cDNA was amplified by conventional PCR in a volume of 20 µL using primers previously described to amplify a product of 723 base pairs (bp) corresponding to the S-segment region and 1,379 bp corresponding to the M-segment region (19). Both reactions were performed using 10 µL of 2×Platinum SuperFi II PCR Master Mix supplemented with 1.5 nm MgCl², 0.3 µm of each primer, and 2 µL of cDNA, and were incubated in a thermocycler according to the standard Platinum SuperFi II cycling.

### Sequencing and phylogenetic analysis

The conventional PCR product was purified with ExoSAP-IT PCR Product Cleanup (Applied Biosystems, California, USA) and sequenced by the automated Sanger method using the SeqStudio Genetic Analyzer platform (Applied Biosystems, California, USA). Consensus sequences were manually produced using the MEGA11-Molecular Evolutionary Genetic Analysis (52).

Complete sequences of Oropouche virus segments S and M were retrieved from the GenBank and comprised two data sets with a total of 101 and 61 sequences, respectively. The alignment was performed using the MUSCLE algorithm (53). The phylogenetic tree was constructed using MEGA11 by the maximum likelihood method using the substitution model Kimura 2-parameter with gamma distribution (K2 + G) for segment S and Tamura 3-parameter with gamma distribution and invariant sites (T92 +G + I) for segment M. The support values were evaluated by bootstrap (1,000 replicates).

### Data analysis

Descriptive analyses were represented through central tendency and dispersion measurements.

## Data Availability

All the Oropouche virus genomes generated and analyzed in this study are available in the GenBank database (NCBI), and the identification accessions can be found in Table S1.

## SUPPLEMENTAL MATERIAL

Table S1 spectrum.01629-23-s0001.pdf *Oropouche orthobunyavirus* S and M segment strains used in this study.

ASM does not own the copyrights to Supplemental Material that may be linked to, or accessed through, an article. The authors have granted ASM a non-exclusive, world-wide license to publish the Supplemental Material files. Please contact the corresponding author directly for reuse.
