# Supplementary material for: Outbreak of Oropouche virus in frontier regions in western Amazon
Source: Microbiol Spectr. 2024 Feb 7;12(3):e01629-23. doi: 10.1128/spectrum.01629-23 (PMC10913433; doi:10.1128/spectrum.01629-23)
Supplement: Table S1 — Oropouche orthobunyavirus S and M segment strains used in this study. [file spectrum.01629-23-s0001.pdf]

Table S1. *Oropouche orhobunyavirus* S and M segment strains used in this study.

Sequence ID	Country	Collection_date	Segment	GenBank
Sample_A01	Brazil: Humaita, AM	16-Nov-2022	S	OR500484
Sample_A03	Brazil: Porto Velho, RO	02-Feb-2023	S	OR500485
Sample_A04	Brazil: Porto Velho, RO	16-Feb-2023	S	OR500486
Sample_A05	Brazil: Porto Velho, RO	14-Feb-2023	S	OR500487
Sample_A06	Brazil: Porto Velho, RO	06-Feb-2023	S	OR500488
Sample_A07	Brazil: Humaita, AM	01-Feb-2022	S	OR500489
Sample_A08	Brazil: Porto Velho, RO	02-Feb-2023	S	OR500490
Sample_A09	Brazil: Humaita, AM	25-Jan-2022	S	OR500491
Sample_A11	Brazil: Porto Velho, RO	08-Mar-2023	S	OR500492
Sample_B05	Brazil: Humaita, AM	16-Nov-2022	S	OR500493
Sample_B11	Brazil: Humaita, AM	16-Nov-2022	S	OR500494
Sample_B12	Brazil: Humaita, AM	16-Nov-2022	S	OR500495
Sample_C05	Brazil: Porto Velho, RO	09-Mar-2023	S	OR500496
Sample_A01	Brazil: Humaita, AM	16-Nov-2022	M	OR500497
Sample_A03	Brazil: Porto Velho, RO	02-Feb-2023	M	OR500498
Sample_A04	Brazil: Porto Velho, RO	16-Feb-2023	M	OR500499
Sample_A05	Brazil: Porto Velho, RO	14-Feb-2023	M	OR500500
Sample_A06	Brazil: Porto Velho, RO	06-Feb-2023	M	OR500501
Sample_A07	Brazil: Humaita, AM	01-Feb-2022	M	OR500502
Sample_A08	Brazil: Porto Velho, RO	02-Feb-2023	M	OR500503
Sample_A09	Brazil: Humaita, AM	25-Jan-2022	M	OR500504
Sample_A11	Brazil: Porto Velho, RO	08-Mar-2023	M	OR500505
Sample_B05	Brazil: Humaita, AM	16-Nov-2022	M	OR500506
Sample_B11	Brazil: Humaita, AM	16-Nov-2022	M	OR500507
Sample_B12	Brazil: Humaita, AM	16-Nov-2022	M	OR500508
Sample_C05	Brazil: Porto Velho, RO	09-Mar-2023	M	OR500509
